# Nitrification and Nitrifying Bacteria in a Coastal Microbial Mat

**DOI:** 10.3389/fmicb.2015.01367

**Published:** 2015-12-01

**Authors:** Haoxin Fan, Henk Bolhuis, Lucas J. Stal

**Affiliations:** ^1^Department of Marine Microbiology, Royal Netherlands Institute for Sea ResearchYerseke, Netherlands; ^2^Department of Aquatic Microbiology, Institute of Biodiversity and Ecosystem Dynamics, University of AmsterdamAmsterdam, Netherlands

**Keywords:** ammonia-oxidation, *amoA*, microbial mat, nitrification, salinity

## Abstract

The first step of nitrification, the oxidation of ammonia to nitrite, can be performed by ammonia-oxidizing archaea (AOA) or ammonium-oxidizing bacteria (AOB). We investigated the presence of these two groups in three structurally different types of coastal microbial mats that develop along the tidal gradient on the North Sea beach of the Dutch barrier island Schiermonnikoog. The abundance and transcription of *amoA*, a gene encoding for the alpha subunit of ammonia monooxygenase that is present in both AOA and AOB, were assessed and the potential nitrification rates in these mats were measured. The potential nitrification rates in the three mat types were highest in autumn and lowest in summer. AOB and AOA *amoA* genes were present in all three mat types. The composition of the AOA and AOB communities in the mats of the tidal and intertidal stations, based on the diversity of *amoA*, were similar and clustered separately from the supratidal microbial mat. In all three mats AOB *amoA* genes were significantly more abundant than AOA *amoA* genes. The abundance of neither AOB nor AOA *amoA* genes correlated with the potential nitrification rates, but AOB *amoA* transcripts were positively correlated with the potential nitrification rate. The composition and abundance of *amoA* genes seemed to be partly driven by salinity, ammonium, temperature, and the nitrate/nitrite concentration. We conclude that AOB are responsible for the bulk of the ammonium oxidation in these coastal microbial mats.

## Introduction

Coastal microbial mats are compact, highly structured, small-scale ecosystems (Stal et al., 1985). These mats are built by cyanobacteria, oxygenic phototrophic bacteria, which through primary production enrich the sediment with organic matter. This organic matter forms the basis of a complex, multi-layered microbial ecosystem. An important process in these microbial mats is the fixation of dinitrogen (N_2_) (Severin and Stal, 2010). N_2_ fixation has been intensively studied in microbial mats, but very little is known about the fate of the fixed nitrogen and about the functioning of the nitrogen cycle in microbial mats. In this study we investigated the oxidation of ammonium and assessed the seasonal variations in microbial mats located along a tidal salinity gradient.

Nitrification is the oxidation of ammonium to nitrate, which occurs in two steps, each carried out by specialist aerobic bacteria (Kowalchuk and Stephen, 2001). The first step, the oxidation of ammonia to nitrite (nitritification), is carried out by two distinct groups of microorganisms: ammonia-oxidizing bacteria (AOB) (two specific groups in beta- and gammaproteobacteria) and ammonia-oxidizing archaea (AOA). The second step is the oxidation of nitrite to nitrate (nitratification) and is carried out by a specialist group of bacteria. No archaea are known to carry out this second reaction. The oxidation of ammonia to nitrite is the rate-limiting step in nitrification. Nitritification is also important because it provides the oxidant for anaerobic ammonium oxidation (anammox) (Jetten et al., 1998). Moreover, nitrite can also be reduced by denitrification (Davidson and Seitzinger, 2006). Both processes eventually lead to the formation of dinitrogen and thus represent a loss of bound nitrogen from the microbial mat ecosystem.

Metagenomic studies (Venter et al., 2004) and the isolation and cultivation of *Nitrosopumilus maritimus* (Könneke et al., 2005), a marine AOA, [now placed within the *Thaumarchaeota* (Brochier-Armanet et al., 2008)] suggested an important role for this group of organisms in ammonia oxidation in the marine environment. This finding challenged the view that bacteria are the main players of microbial ammonia oxidation and has led to a large volume of research for the presence of AOA and AOB in a wide range of ecosystems. The presence of ammonia oxidizers is usually determined through the detection of *amoA*, the gene encoding the alpha subunit of ammonia monooxygenase, an enzyme that performs the first step in ammonia oxidation in both AOA and AOB. The ecological importance of AOA and AOB in nitrification has been determined in several studies. On the one hand, some studies reported that the archaeal *amoA* genes outnumbered those of bacteria by orders of magnitudes (as was the case for example in the North Atlantic Ocean and in the North Sea (Wuchter et al., 2006), in Monterey Bay and near Hawaii (Mincer et al., 2007), and in several estuaries (Caffrey et al., 2007). On the other hand, some studies reported that bacterial *amoA* genes were more abundant than the archaeal *amoA* (Mosier and Francis, 2008; Christman et al., 2011). Since also gene or cell abundance do not necessarily reflect activity, the relative contribution of AOA and AOB to ammonia oxidation in coastal sediments remains uncertain. There is however good evidence for different niches for AOA and AOB. The former possesses a high affinity (low *K*_m_) for ammonia and therefore seems to particularly dominate environments that are very low in it, while the latter seems to prefer environments with high ammonia concentrations (Martens-Habbena et al., 2009). Compared to many terrestrial and marine environments, the ecology of ammonia oxidizer communities and their role in nitrification in coastal microbial mats have been poorly studied. AOA and AOB may be subject to different selection pressures that result from biotic and abiotic conditions and the different physiology that characterizes these organisms. A suite of environmental parameters may control nitrification in coastal sediments. These include besides ammonia, oxygen- and sulfide concentrations, the rate of carbon metabolism, and the presence or absence of vegetation or macro-fauna (Herbert, 1999). Coastal microbial mats harbor a multitude of potential environmental niches as the result of the large daily fluctuations of the key geochemical parameters such as: oxygen, pH, and sulfide (Revsbech et al., 1983).

The aims of this study were to identify the ammonia oxidizing communities in the three types of microbial mats and to elucidate the factors that determine the abundance and activity of ammonia oxidizers. Therefore, we measured the potential rate of nitrification and investigated the diversity and abundance of *amoA* for AOB and AOA in three different mat types during four different seasons. We monitored the key environmental variables and linked them to changes in ammonia oxidizer communities and their activities (*amoA* gene transcripts).

## Materials and methods

### Sampling

The study site was located on the North Sea beach of the Dutch barrier island Schiermonnikoog. The geographical locations and descriptions of the three types of microbial mats (stations) that were sampled during this study as well as the vegetation and primary cyanobacterial species at these stations are presented in Table 1. The stations were located along a transect perpendicular to the beach covering the tidal gradient. Sampling was done five times during 2010 and 2011 to cover the four seasons. Samples were taken from the top 25–30 mm of the mat using custom-made transparent Lexan cylinder corers of 50 mm inner diameter and 60 mm height. The cores were transported back to the laboratory within 4 h of sampling and subsequently kept at ambient temperature and light. Incubation experiments for measuring the potential nitrification rate started within 24 h after sampling. Additional samples were taken from the natural mats for nucleic acid extraction. These samples were taken from the top 10 mm of the mat by using as a corer a 10-ml syringe from which the needle connector was removed. These mat samples were divided into four equal parts using a scalpel, put into cryo-vials, and immediately frozen in the field in liquid nitrogen.

**Table 1:** **The geographical coordinates and description of the mats investigated in this study**.

**Station**	**Geographical coordinates**	**Description**	**Vegetation**	**Dominant cyanobacterial species**
Station I	53°29.445′N, 6°8.718′E	Mainly freshwater influenced site, close to the dunes. Irregularly inundated	*Elymus arctus*	*Nostoc*
			*Juncus gerardi*	*Calothrix*
			*Glaux maritima*	*Anabaena*
			*Ammophila arenaria*	*Spirulina*
			*Scirpus maritimus*	*Nodularia*
				*Synechocystis*
				*Merismopedia*
				*Gloeocapsa*
Station II	53°29.460′N, 6°8.309′E	Seawater influenced site, developing microbial mat. At the low water mark	No vegetation	*Lyngbya Leptolyngbya*
Station III	53°29.445′N, 6°8.342′E	Seawater and freshwater influenced site, located between St1 and St2, at the edge of the salt marsh	*Salicorni*a sp. *Puccinellia distans*	*Microcoleus Lyngbya*

*The cyanobacteria were identified by light microscopy based on their typical morphological characteristics*.

### Chemical analyses

For nutrient analyses 5 g mat sample (top 10 mm) was extracted with 40 ml 2 *M* KCl. The extracts were filtered through Whatman GF/F filters and the filtrates were kept at −20°C until analysis (within a month). Nutrient (DIN and phosphate) concentrations were measured by a standard colorimetric method using an automated Segmented Flow Analyzer. Other mat samples were freeze-dried for the determination of total nitrogen (TN), total organic carbon (TOC) and C/N ratio by EA-IRMS (DELTA V Advantage; Thermo Fisher Scientific, Bremen, Germany).

### Potential nitrification rate

The potential rate of nitrification was determined by using the ^15^N isotope dilution method by the addition of ^15^N-labeled nitrate (Kirkham and Bartholomew, 1954). The measurements were performed in triplicate in the intact sediment cores. The ^15^N nitrate solution was injected into the sediment core. The needle was inserted fully into the sediment core and the syringe plunger was depressed while the needle was withdrawn out of the sediment as to distribute the label equally in the sediment. Four injections were made in each sediment core. Three cores from each station were frozen (−20°C) immediately after the injection. The other cores were incubated at *in situ* temperature (Table 2) under a 12–12 h light-dark cycle for 24 h. Subsequently, the inorganic nitrogen was extracted from the cores by 2 *M* KCl. The nitrogen isotopic composition of ${\text{NO}}_{\text{x}}^{-}$ was determined using the ammonia diffusion procedure according to Gribsholt et al. (2005). Briefly, to 60 ml GF/F (Whatman) filtered extract 0.1 g NaCl and 300 mg MgO was added to convert ${\text{NH}}_{4}^{+}$ to NH_3_. The NH_3_ was trapped on an acidified (H_2_SO_4_) 10 mm GF/D filter packet floating on the surface. After 8 days shaking at room temperature, the filter was removed. Subsequently, Devarda's Alloy (75 mg) was added to convert ${\text{NO}}_{2}^{-}+{\text{NO}}_{3}^{-}$ to NH_3_, which was collected on a new acidified filter. The filters were dried for 2 days in an exicator and analyzed using a Flash EA-1112 series elemental analyzer coupled in-line via a conflo II interface with a Delta S isotope ratio mass spectrometer (EA-IRMS, Thermo Fisher Scientific, Bremen, Germany). The rate of nitrification was calculated according to the equation of Norton and Stark (2011).

**Table 2:** **Physicochemical parameters in the microbial mats during the 2010–2011 sampling period**.

**Temperature (°C, sediment)**	**July (2010)**	**September (2010)**	**November (2010)**	**January (2011)**	**April (2011)**
	**17**	**10**	**9**	**0**	**8**
**ST1**					
${\text{NH}}_{4}^{+}$(μmol/l)	128.9 ± 3.0	83.7 ± 17.3	252.4 ± 25.6	191.3.5 ± 23.4	233.1 ± 23.7
NO${}_{\text{X}}^{-}$(μmol/l)	23.3 ± 4.6	8.4 ± 1.1	11.4 ± 2.1	9.6 ± 4.6	25.9 ± 3.4
TOC(%)	0.04	0.06	0.04	0.04	0.04
TN(%)	0.006	0.01	0.006	0.007	0.007
C/N	6.7	6.0	6.7	5.7	5.7
Salinity(psu)	18	19	15	15	17
**ST2**					
NH4 ^+^(μmol/l)	587.9 ± 41.2	216.2 ± 69.8	1094 ± 91.3	736.8 ± 199.6	486.1 ± 61.3
NO_X_-(μmol/l)	22.4 ± 14.7	8.7 ± 1.4	9.8 ± 3.2	6.2 ± 1.1	20.6 ± 4.5
TOC(%)	0.19 ± 0.01	0.20 ± 0.03	0.13 ± 0.02	0.20 ± 0.03	0.17 ± 0.02
TN(%)	0.03	0.04	0.02	0.03	0.03
C/N	6.3	5.0	6.5	6.7	5.7
Salinity(psu)	28	28	29	30	28
**ST3**					
NH_4_+(μmol/l)	217.3 ± 102.3	255.9 ± 68.4	782.6 ± 158.5	510.2 ± 62.2	475.8 ± 3.5
NO_X_-(μmol/l)	16.0 ± 3.7	7.6 ± 1.9	6.6 ± 1.9	2.8 ± 0.6	19.6 ± 4.0
TOC(%)	0.11 ± 0.03	0.15 ± 0.02	0.18 ± 0.02	0.15 ± 0.02	0.13 ± 0.02
TN(%)	0.02	0.03	0.03	0.03	0.02
C/N	5.5	5.0	6.0	5.0	6.5
Salinity(psu)	25	25	23	22	23

*TOC, total organic carbon; TN, Total nitrogen*.

### Nucleic acid extraction and geochip analysis

DNA and RNA were extracted using the MoBio UltraCLEAN soil DNA kit and the RNA PowerSoil® Total Isolation Kit, respectively (MoBio Laboratories, Inc., Carlsbad, CA, USA) according to the manufacturer's instructions. The quantity and quality were determined and checked by Nanodrop (Nanodrop ND1000, Thermo Scientific, Wilmington, DE, USA) and agarose gel electrophoresis, respectively. The RNA extracts were immediately treated with RNase free DNase I (Deoxyribonuclease I, Amplification Grade, Invitrogen Corporation, Carlsbad, CA, USA). Remaining DNA contamination of the RNA extracts was checked by PCR using the RNA extract as a template. RNA concentration and quality were checked again as described above. The DNA-free RNA was reverse transcribed to copy DNA using Superscript II Reverse Transcriptase and random primers (Invitrogen Corporation, Carlsbad, CA, USA) following the manufacturer's manual. Two controls were performed that either lacked reverse transcriptase or RNA. PCR reactions were performed to check the transcription to cDNA and controls were included as described above. The synthesized cDNA was kept at −20°C until further use.

In addition, we used a functional gene microarray system, the GeoChip (Tu et al., 2014), containing probes for genes involved in the majority of important biogeochemical nutrient cycles. For this study, we extracted data from the GeoChip for *amoA* genes (~1340 probes). We analyzed DNA samples from July 2010 and January 2011 by the GeoChip 4.2. DNA was extracted from triplicate samples from each of the three types of microbial mats. The DNA was purified using UltraClean 15 DNA purification Kit (MoBio Laboratories, Inc., Carlsbad, CA, USA) in order to achieve the quality necessary for hybridization on the chip. The DNA quantity was measured using the Nanodrop ND-1000 system. The procedures for DNA labeling and microarray hybridization followed previously established protocols (Wu et al., 2006). Briefly, 800 ng DNA was labeled with fluorescent Cy-5 dye by random priming and re-suspended in 50 μl hybridization solution (40% formamide, 5 × SSC, 5 μg of unlabeled herring sperm DNA (Promega, Madison, WI), and 0.1% SDS) and 2 μl universal standard DNA (0.2 pmol μl^−1^) labeled with the fluorescent Cy-3 dye (Liang et al., 2010), denatured for 5 min at 95°C and maintained at 50°C until loaded onto the microarray slides. Arrays were hybridized on a MAUI Hybridization Station (Roche, South San Francisco, CA) for 12 h at 42°C. The hybridized microarrays were scanned by a ScanArray Express (Perkin- Elmer, Wellesley, MA) at 95% laser power and 85% photomultiplier tube gain. The resulting images were analyzed by ImaGene with signals processed as SN>2.0 (signal to noise ratio).

### Quantitative PCR (qPCR) analysis

qPCR analyses were run on a Corbett Rotor-Gene 6000 TM (Corbett Life Science, Sydney, Australia). The copy numbers of AOB and AOA were determined by primers amoA-1F and amoA-2R (Tm = 53°C) (Rotthauwe et al., 1997) and by CrenAmoAQ-F (Mincer et al., 2007) and Arch-AmoA-R (Tm = 51°C) (Francis et al., 2005), respectively. We determined the gene copy number in the mat samples in triplicate. Standard curves were made by dilution series of linearized plasmids (quantified by Nanodrop before using as standard for quantification) containing the target genes and were run in parallel with each analysis as well as with non-template controls. The reaction mixture (15 μl) contained 7.5 μl of Absolute™ QPCR SYBR® Mix (Thermo Fisher Scientific, Rockford, IL, USA), 0.2 pmol/μl primers, 1 μl template and sterilized MQ water. Cycling conditions were as follows: 95°C 15 min, 45 cycles of 15 s 95°C, 20 s Tm, and 20 s at 72°C, followed by melting curve analysis (50–95°C). The standard curves spanned a range from 15 to 1.7 × 10^6^ copies per μl for the β-AOB and 2.2 to 1.2 × 10^6^ copies per μl for the AOA. PCR efficiencies (E) and correlation coefficients for β-AOB were 78–88% and *R*^2^ = 0.99 and for AOA were 85–98% and *R*^2^ = 0.99.

### Sequences and statistical analysis

Ammonia monooxygenase alpha subunit amino acid sequences obtained from GeoChip hybridization (50-mer oligonucleotide probes) and some reference sequences retrieved from GenBank were used to produce neighbor-joining trees and the reliability of the phylogenetic reconstructions was evaluated by bootstrapping (1000 replicates) using MEGA 6 (Molecular Evolutionary Genetics Analysis, http://www.megasoftware.net) (Tamura et al., 2013).

In order to summarize the shared genes at station and season level, the genes detected in the three replicates of each station from July 2010 and January 2011 by the GeoChip were deployed as one pool (mean value from the three replicates). The determination of the shared genes, unique genes and diversity indices was done using an online pipeline (http://ieg.ou.edu/). The proportion of shared genes of two stations was calculated as the number of shared genes divided by the total number of genes detected in these stations. The proportion of unique genes at each station was calculated as the number of unique genes at each station divided by the total number of genes detected at that station.

Cluster analysis, multi-response permutation procedure (MRPP) and canonical correspondence analysis (CCA) (see below) were made based on the community data from the GeoChip. Cluster analysis of the community composition was done using PAST (http://folk.uio.no/ohammer/past/). MRPP (Bonferroni-corrected) using Bray-Curtis distance was used to test for significant differences in community composition. The MRPP was carried out in the open source software R (Team, 2011), using vegan package (Oksanen, 2011). The MRPP A-statistics describes the within and between group relatedness relative to what is expected by chance. A *p* < 0.05 and an A-statistics >0.1 is considered to be a significant difference between groups (McCune et al., 2002). In order to test the relationship between the community composition and the environmental variables, CCA was carried out using Canoco 4.5 for Windows (ter Braak, 1989). The significance of the whole canonical model was tested by 999 permutations. One-way ANOVA, correlation test (Spearman and Pearson), and stepwise regression were carried out in SigmaPlot (Version12). Stepwise regression was carried out to test the influence of the environmental factors and abundance of *amoA* gene and transcript on potential nitrification rates.

## Results

### Phylogeny of *amoA*

**Figure 1:**
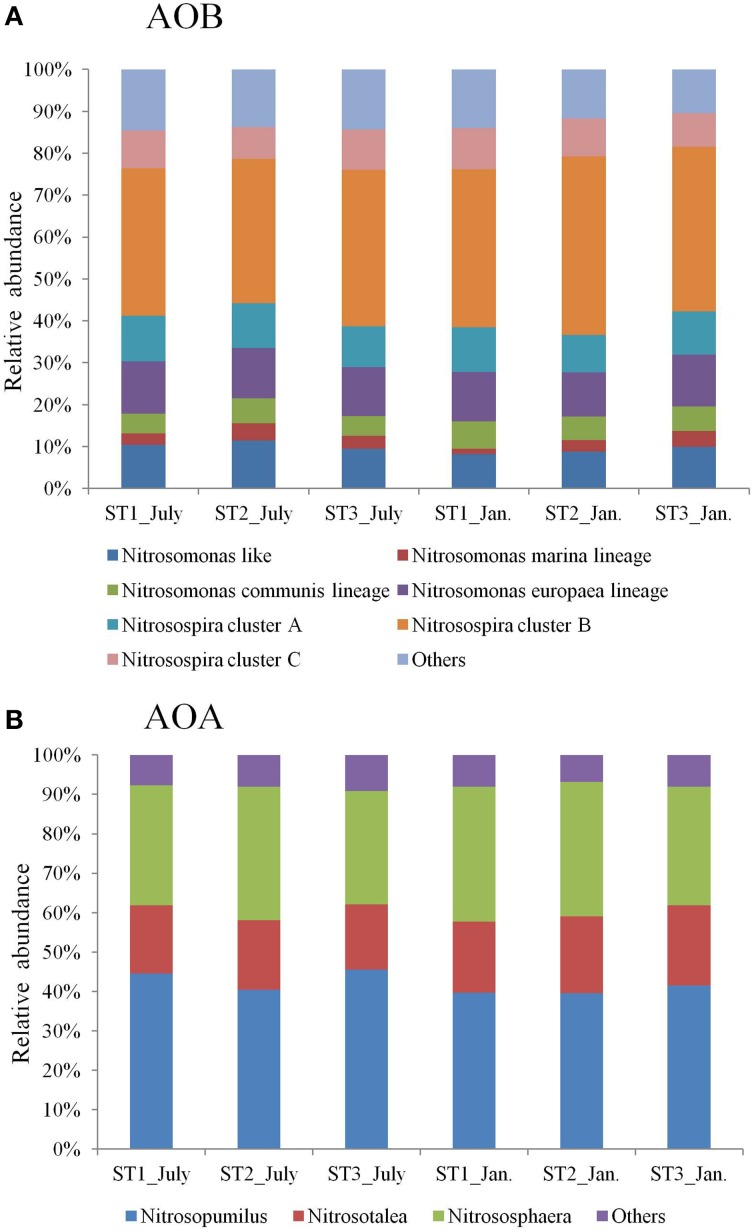
**Relative abundance of (A) probes affiliated with the major bacterial ***amoA*** clusters suggested by Purkhold et al. (2003) and Francis et al. (2003) in the analyzed microbial mats; (B) probes affiliated with the major archaeal ***amoA*** clusters suggested by Pester et al. (2012) in the analyzed microbial mats**.

In all microbial mats, AOB *amoA* genes were detected belonging to Beta- and Gammaproteobacteria (Figure 1A). The majority (>98% signal intensities) AOB *amoA* sequences retrieved from the GeoChip hybridizations belonged to Betaproteobacteria (β-AOB). Among the Betaproteobacteria, the sequences clustered with *Nitrosospira* and *Nitrosomonas*. The sequences belonging to *Nitrosospira* can be subdivided into three major clusters (Table 3). Several cultivated species, such as *Nitrosospira multiformis* (AAC25057), *Nitrosospira briensis* and *Nitrosospira* sp. REGAU (AAV34189) were detected. 8.0% of the sequences obtained from the GeoChip belong to *Nitrosospira* cluster C. *Nitrosovibrio* sp. FJ182 (ABB69924) and some uncultured clones were detected that grouped in cluster C. The sequences belonging to *Nitrosomonas* can be sub-divided into four major clusters (Table 3). The nomenclature suggested by Purkhold et al. (2003) and Francis et al. (2003) was used. At all stations, the shifts between different groups were minor. Phylogenetic analysis revealed a broad distribution of archaeal *amoA* genes (Figure 1B). The nomenclature suggested by Pester et al. (2012) was used. 41.1, 14.7, 37.9, and 6.3% of the total number of sequences obtained from the GeoChip fell into *Nitrosopumilus, Nitrosotalea, Nitrososphaera*, and others, respectively (Table 3). Based on the GeoChip signal intensities, *Nitrosopumilus* and *Nitrososphaera* were predominant and accounted for respectively 39.6–45.5% and 28.7–34.3% of total AOA *amoA* gene signal intensities. *Nitrosotalea* accounted for 16.5–20.3% of the total AOA amoA gene signal intensities. Other groups accounted for the remaining 6.9–9.3% of the total AOA amoA gene signal intensities. In none of the stations shifts between different groups were observed.

**Table 3:** **Summary of the percentage of different types of ***amoA*** detected at each station**.

	**ST1_July (%)**	**ST2_July (%)**	**ST3_July (%)**	**ST1_Jan. (%)**	**ST2_Jan. (%)**	**ST3_Jan. (%)**
**AOB**						
*Nitrosomonas* like	10.3	11.4	9.5	8.3	8.8	9.9
*Nitrosomonas marina* lineage	2.8	4.1	3.1	1.2	2.8	3.8
*Nitrosomonas communis* lineage	4.6	6.0	4.7	6.6	5.6	5.8
*Nitrosomonas europaea* lineage	12.5	11.9	11.7	11.8	10.5	12.4
*Nitrosopira* cluster A	10.9	10.7	9.7	10.7	9.0	10.3
*Nitrosopira* cluster B	35.2	34.5	37.4	37.7	42.6	39.3
*Nitrosopira* cluster C	9.1	7.5	9.6	9.8	9.0	8.0
Others	14.6	13.8	14.4	14.0	11.7	10.5
**AOA**						
*Nitrosopuilus*	44.5	40.4	45.5	39.7	39.6	41.5
*Nitrosotalea*	17.3	17.7	16.5	17.9	19.4	20.3
*Nitrososphaera*	30.5	33.9	28.7	34.3	34.1	30.0
Others	7.7	8.1	9.3	8.0	6.9	8.1

*The percentages were calculated by dividing the hybridization intensity of each gene type on the GeoChip by the total signal intensity of all AOB or AOA genes detected on the array*.

### AOB and AOA diversity and community composition

The diversity of AOA and AOB *amoA* genes revealed by the GeoChip analysis is summarized in Table 4. The GeoChip detected a total of 112 AOB *amoA* sequences. The average number of AOB *amoA* sequences at Station 1 (July), Station 2 (July), Station 3 (July), Station 1 (January), Station 2 (January), and Station 3 (January) was 103, 94, 62, 85, 68, and 49, respectively (Table 4). Fourteen sequences were unique, meaning that they were detected only at one station and in one season. In July, Station 1 harbored 9 unique sequences, which was 8.7% (9/103) of the total number detected. Station 2 and Station 3 harbored 3.2% (3/94) and 1.6% (1/62) unique sequences, respectively. In January, the number of unique sequences in Station 1 dropped to 1, which was only 1.2% (1/85) of the total number detected. No unique sequences were observed in January at Station 2 and Station 3. Pairwise comparison of AOB *amoA* sequences showed a high number of shared AOB *amoA* sequences between July and January as well as between the stations: 79.1% (Station 1 July and January), 68.8% (Station 2 July and January), 70.8% (Station 3 July and January), 61.3–83.0% (Station 1 and Station 2), 44.8–57.5% (Station 1 and Station 3), and 52.1–62.5% (Station 2 and Station 3).

**Table 4:** **Summary of AOB and AOA ***amoA*** detected by GeoChip, including the number and percentage of shared (italic) and unique (bold) sequences, and the diversity indices for each station**.

	**ST1_July**	**ST2_July**	**ST3_July**	**ST1_Jan**.	**ST2_Jan**.	**ST3_Jan**.
**AOB**						
ST1_July	**9(8.74%)**	*88(82.95%)*	*59(57.48%)*	*83(79.05%)*	*65(61.32%)*	*47(44.76%)*
ST2_July		**3(3.19%)**	*60(62.50%)*	*77(75.49%)*	*66(68.75%)*	*49(52.13%)*
ST3_July			**1(1.61%)**	*54(58.06%)*	*51(64.56%)*	*46(70.77%)*
ST1_Jan.				**1(1.18%)**	*64(77.91%)*	*46(50.56%)*
ST2_Jan.					**0(0.00%)**	*46(64.79%)*
ST3_Jan.						**0(0.00%)**
Richness^*^	103	94	62	85	68	49
Shannon-Weaver	4.8	4.7	4.3	4.7	4.5	4.2
**AOA**						
ST1_July	**5(6.3%)**	*69(78.4%)*	*52(61.2%)*	*68(77.3%)*	*53(61.6%)*	*46(54.1%)*
ST2_July		**3(3.9%)**	*56(71.8%)*	*65(73.9%)*	*57(72.2%)*	*50(64.1%)*
ST3_July			**1(1.8%)**	*51(62.2%)*	*47(68.1%)*	*44(68.8%)*
ST1_Jan.				**4(5.3%)**	*57(73.1%)*	*49(62.8%)*
ST2_Jan.					**0(0.00%)**	*47(74.6%)*
ST3_Jan.						**1(2.0%)**
Richness^*^	80	77	57	76	59	51
Shannon-Weaver	2.9	2.9	2.7	2.9	2.7	2.7

* *Richness is determined as probe numbers detected*.

We detected 95 AOA *amoA* sequences in the mats. The highest number of AOA *amoA* sequences was detected in July at Station 1 (80). We detected 77, 57, 76, 59, and 51 AOA *amoA* sequences at Station 2 (July), Station 3 (July), Station 1 (January), Station 2 (January), and Station 3 (January), respectively (Table 4). A considerable number of AOA *amoA* sequences were also shared between stations and seasons (Table 4). The highest Shannon index was observed at Station 1 and the lowest was at Station 3, irrespective the time of sampling.

**Figure 2:**
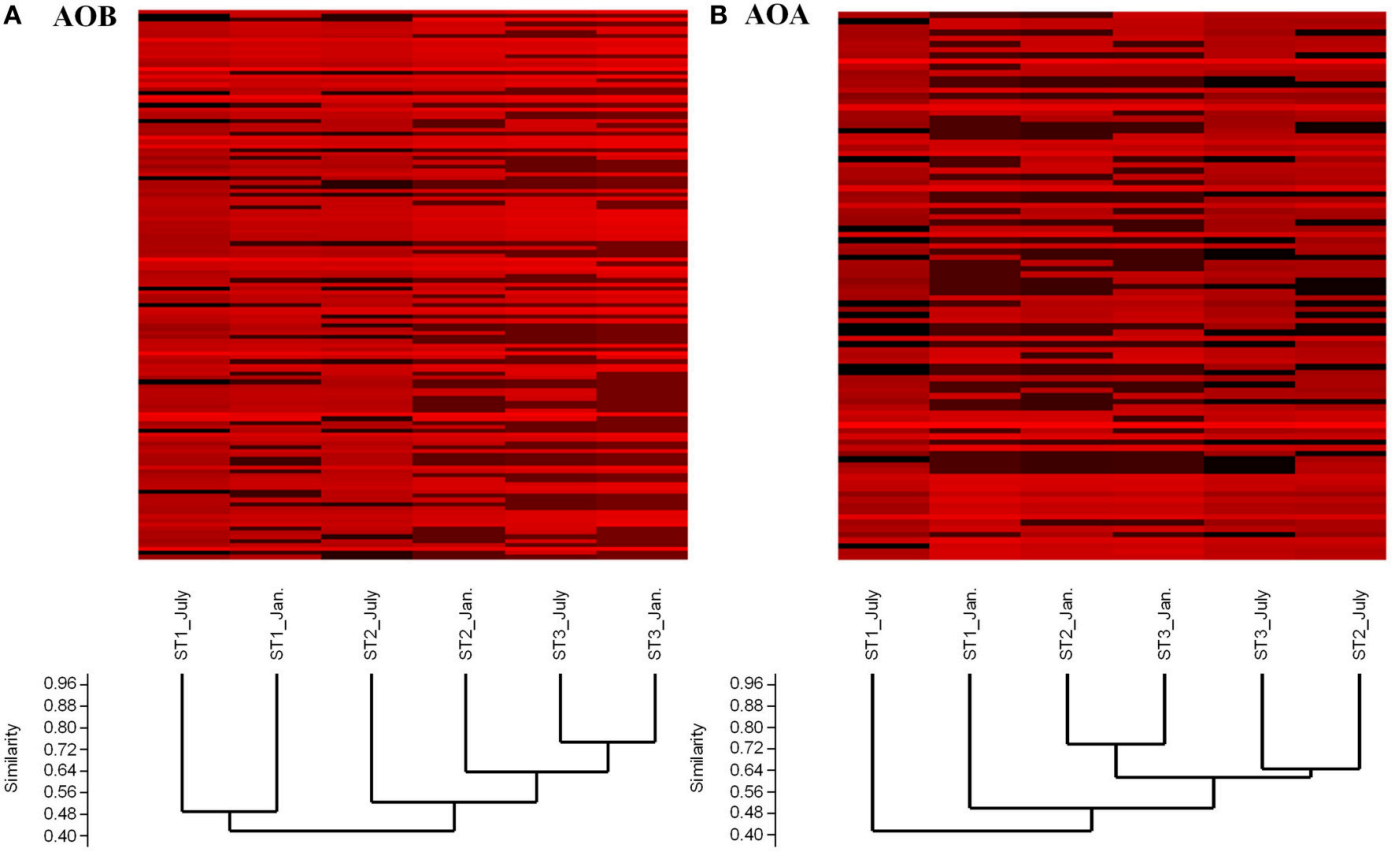
**Cluster analysis of ***amoA*** community composition at the different sampling stations. (A)** Cluster analysis of betaproteobacterial *amoA* gene composition **(B)** Cluster analysis of archaeal *amoA* gene composition. Sample codes consist of the sampling station and the month of sampling.

Cluster analysis revealed that the AOB community composition (based on the *amoA* gene diversity) was largely the same in summer and winter in each of the Stations 1 and 3. Station 2 and Station 3 clustered and were dissimilar from Station 1 (Figure 2A). A similar distribution was also found for the AOA community: Station 2 and Station 3 were more similar and separated from the community of Station 1. In addition, the July AOA community composition was separated from that of January in the Stations 2 and 3 (Figure 2B).

MRPP statistics was carried out to test the differences of the AOB and AOA *amoA* composition between the stations and seasons (July and January) based on the GeoChip data. When the data from July or January were analyzed individually, no significant differences were observed between stations. When the data from the two seasons were combined, distinct communities of AOB and AOA were found in Station 1 when compared to stations 3 (*p* < 0.05) (Table 5). Stations 2 and 3 did not show significant different ammonia-oxidizer communities nor did station 1 differ from station 2 (*p* > 0.05) (Table 5). There were no seasonal differences in the ammonia-oxidizer communities in any of the stations (data not shown).

**Table 5:** **Multi-response permutation procedure A-values of the ammonia oxidizing community composition**.

**Difference between group**	
**Spatial differences**	**A-value**
**AOA**	
ST1 vs. ST2	0.135 (*p* = 0.096)
ST1 vs. ST3	0.3568 (*p* = 0.012)^*^
ST2 vs. ST3	0.065 (*p* = 0.285)
**AOB**	
ST1 vs. ST2	0.135 (*p* = 0.09)
ST1 vs. ST3	0.3645 (*p* = 0.015)^*^
ST2 vs. ST3	0.04 (*p* = 0.495)

* *Means p < 0.05 (statistical difference between AOA and β-AOB amoA profiles assessed using multi-response permutation procedure)*.

### AOB and AOA abundance and activity

Abundance of archaeal and bacterial *amoA* genes was quantified in three stations from different seasons. Because of a negligible signal for nitrifiers belonging to the Gammaproteobacteria, we focus on β-AOB. β-AOB *amoA* genes were detected in all three stations and in all seasons except at Station 1 in September. The numbers ranged from below detection level to 1.7 × 10^7^ copies g^−1^ (sediment) (Table 6). The highest number of β-AOB *amoA* genes was always observed in January at all stations. AOB *amoA* gene was undetectable in September and April at Station 1. The lowest β-AOB *amoA* gene abundance at Stations 2 and 3 was observed in November and April, respectively. β-AOB *amoA* gene abundance increased from the station at the dunes to the low water mark in three of the four seasons tested, the exception was that in November the highest value was found in the intertidal Station 3. AOA *amoA* copies ranged from below detection level to 1.2 × 10^4^ copies g^−1^ sediment. AOA *amoA* was undetectable at Station 1, irrespective of the season, and in April at Station 3. The highest number of AOA *amoA* copies was detected in January at Station 2. The β-AOB *amoA* copy number was significantly (*p* < 0.05) higher in all samples except in September at Station 1 when neither β-AOB nor AOA *amoA* genes were detected. β-AOB *amoA* abundance was significantly correlated (Spearman, *r* = 0.70, *p* < 0.01) with AOA *amoA* abundance.

**Table 6:** **Potential nitrification rate (PNR) (μmol N m^−2^ d^−1^) and abundance of ***amoA*** (of β-AOB and AOA) and their transcripts in the microbial mats during the 2010–2011 sampling period (copies g^−1^ sediment)**.

	**July**		**September**		**November**		**January**		**April**	
		**SD**		**SD**		**SD**		**SD**		**SD**
**STATION 1**										
PNR	n.d.		43.5	15.6	463.7	103.7	356.9	86.3	248.7	27.0
AOB DNA	550	100	n.d.		99		3.4 × 10^5^	5.9 × 10^4^	n.d.	
AOB cDNA	86	150	2.0 × 10^3^	1.7 × 10^3^	n.d.		290	510	970	390
AOA DNA	n.d.		n.d.		n.d.		n.d.		n.d.	
AOA cDNA	n.d.		n.d.		n.d.		n.d.		n.d.	
**STATION 2**										
PNR	102.8	23.6	33.6	12.8	274.9	17.8	327.8	96.4	97.5	55.4
AOB DNA	8.2 × 10^6^	5.8 × 10^6^	2.3 × 10^5^	1.2 × 10^5^	1.4 × 10^5^	5.6 × 10^4^	1.7 × 10^7^	6.2 × 10^6^	1.8 × 10^6^	8.5 × 10^5^
AOB cDNA	n.d.		360	250	3.9 × 10^3^	2.2 × 10^3^	n.d.		n.d.	
AOA DNA	460	390	210	66	150	340	1.0 × 10^4^	4.6 × 10^3^	5.9 × 10^3^	55
AOA cDNA	n.d.		n.d.		n.d.		n.d.		n.d.	
**STATION 3**										
PNR	177.4	15.2	57.5	31.7	300.3	56.8	537.0	99.2	237.7	86.4
AOB DNA	1.1 × 10^5^	2.6 × 10^4^	5.4 × 10^5^	3.3 × 10^5^	3.8 × 10^6^	1.2 × 10^6^	4.9 × 10^6^	1.9 × 10^6^	8.9 × 10^3^	2.5 × 10^3^
AOB cDNA	n.d.		830	140	3.5 × 10^3^	2.1 × 10^3^	1.6 × 10^3^	830	1.4 × 10^3^	180
AOA DNA	330	2.2 × 10^3^	4.3 × 10^3^	2.8 × 10^3^	110	47	1.7 × 10^3^	800	n.d.	
AOA cDNA	n.d.		n.d.		n.d.		n.d.		n.d.	

*SD, standard deviation; n.d., below detection*.

The expression of β-AOB *amoA* was detected in four of the 5 months at Station 1 (undetectable in November) and Station 3 (undetectable in July) with highest expression in April and November, respectively. At Station 2, the β-AOB *amoA* expression was only detected in September and November. Gene expression of AOA *amoA* was below the limit of detection in all samples.

### Relationship of ammonia oxidizer community with environmental variables

**Figure 3:**
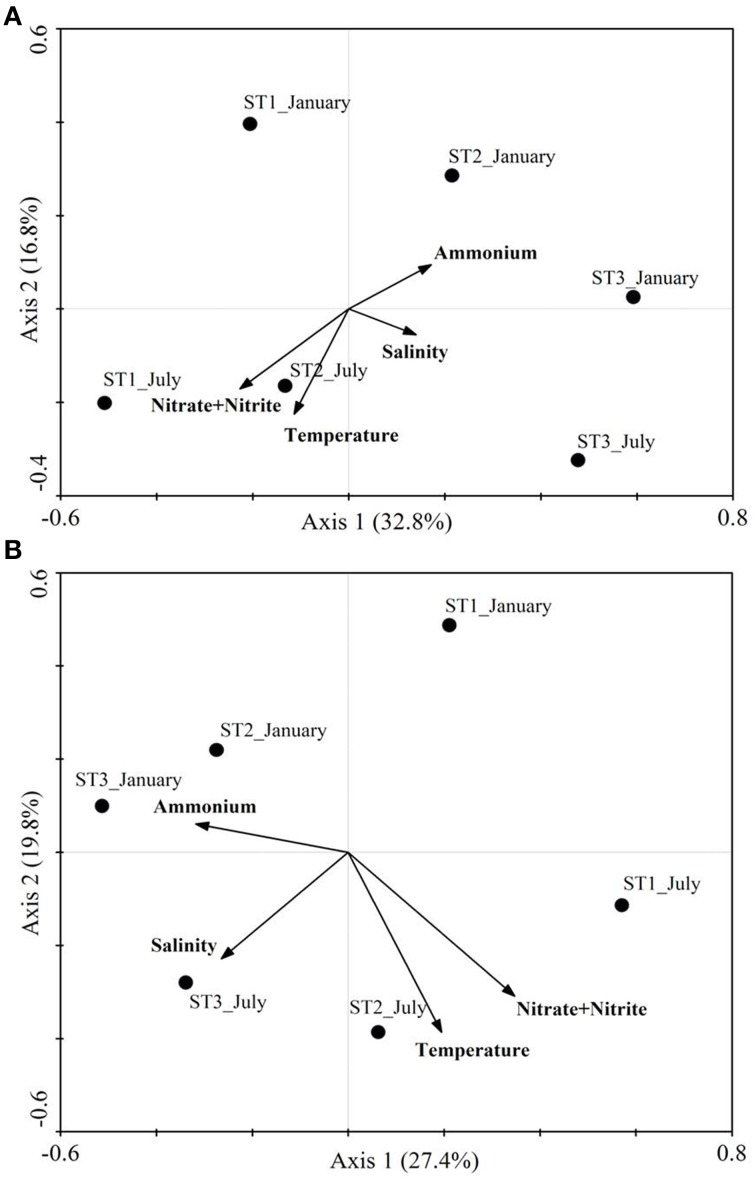
**Canonical correspondence analysis of ***amoA*** community composition of mat samples. (A)** β-AOB *amoA* gene, **(B)** AOA *amoA* gene. Points represent the *amoA* gene community from seasonal samples at the indicated station. Arrows represent the relationship between environmental parameters with the *amoA* communities.

Canonical correspondence analysis showed that ammonia oxidizer (AOA and AOB) communities were significantly correlated with salinity, temperature, ammonium and nitrate content (based on 999 Monte Carlo permutations, *p* = 0.001) (Figure 3). The variables in the first and second axes explained 49.6 and 47.2% of the total variation of AOB and AOA composition, respectively.

Environmental variables influenced also the abundance of ammonia oxidizers. Across all samples, both β-AOB (Spearman, *r* = 0.59, *p* < 0.01, *n* = 15) and AOA (Spearman, *r* = 0.73, *p* < 0.01, *n* = 15) *amoA* abundance were positively correlated with salinity and organic matter (AOB: Spearman, *r* = 0.87, *p* < 0.01, *n* = 15; AOA: Spearman, *r* = 0.60, *p* < 0.05, *n* = 15). Abundance of β-AOB *amoA* gene also correlated with ammonium content (Spearman, *r* = 0.78, *p* < 0.05, *n* = 15). No significant correlation was found between abundance of ammonia oxidizers and other environmental variables (measured in this study).

### Potential nitrification rate (PNR)

**Figure 4:**
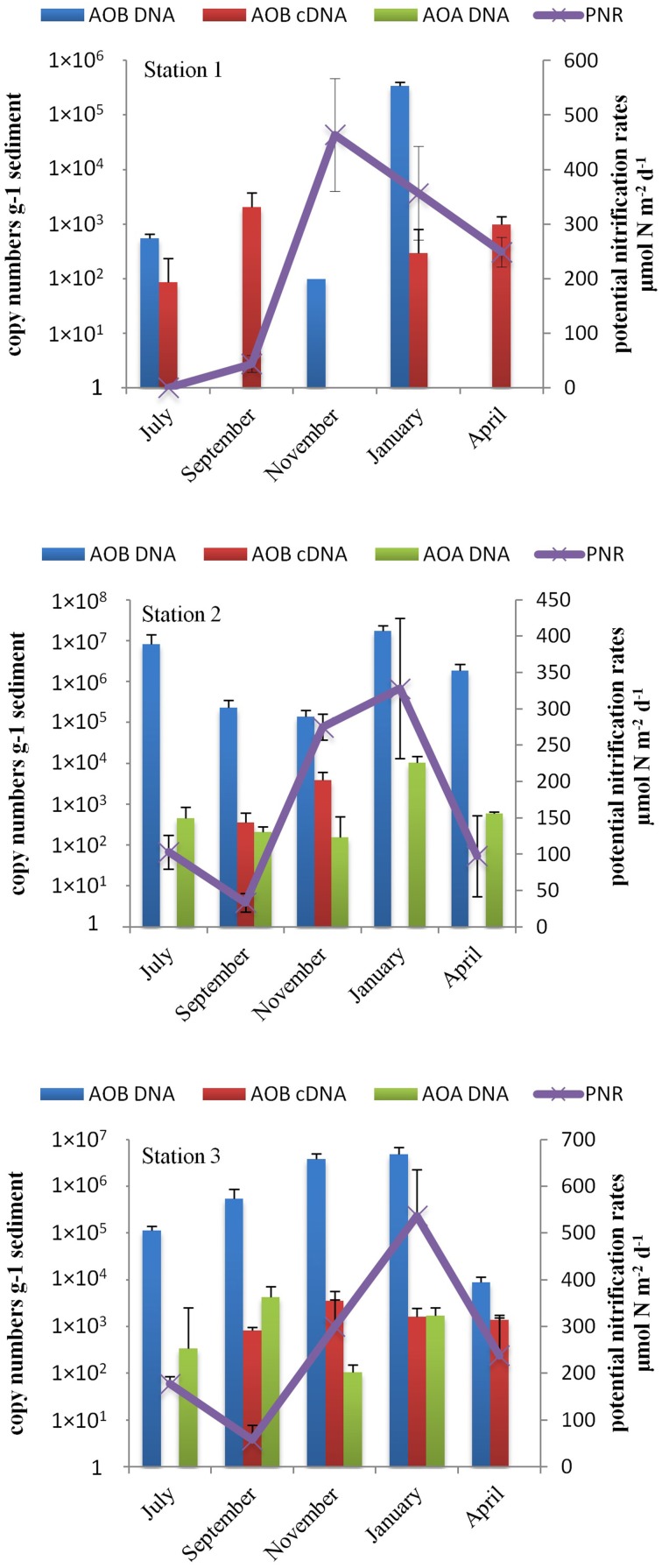
**Mean (±standard error, ***n*** = 3) potential nitrification rate (PNR) and abundance of *amoA* (of β-AOB and AOA) and their transcripts at Station 1, Station 2, and Station 3 during the 2010–2011 sampling period (July, September, November, January, and April)**.

The potential nitrification rate was measured using the isotope (^15^N nitrate) dilution method (Kirkham and Bartholomew, 1954) and the measurements covered the four seasons during 2010 and 2011. The potential rate of nitrification varied between stations as well as between seasons and ranged from 34 to 537 μmol N m^−2^d^−1^ (Table 6). At all three stations the potential rate of nitrification showed a similar seasonal pattern with higher rates occurring in November and January and lowest rates occurring in July and September (Figure 4). The highest rate was measured at Station 3 (537 μmol N m^−2^d^−1^) while the lowest was detected at Station 2. The potential rate of nitrification was always lowest at Station 2, irrespective of the season.

## Discussion

The GeoChip analyses revealed that ammonia-oxidizing bacteria and archaea are common in the microbial mats of the North Sea barrier island Schiermonnikoog. AOB and AOA *amoA* genes that were detected by GeoChip were distributed throughout the phylogenetic trees of AOB and AOA *amoA*. The AOB *amoA* sequences in the microbial mat were dominated by clusters B and C, which both relate to *Nitrosospira* cluster 3, first described by Purkhold et al. (2003). This is consistent with what other studies reported. *Nitrosospira* cluster 3 is found predominantly in brackish and marine environments (Francis et al., 2003; Bernhard et al., 2005; Freitag et al., 2006; Wankel et al., 2011). A considerable number of sequences was found that belong to *Nitrosomonas*. The AOB in *Nitrosomonas* spanned a wide range of physiological types and inhabit oligohaline to polyhaline environments (Koops and Pommerening-Roser, 2001; Francis et al., 2003). The AOA *amoA* sequences detected in the microbial mats investigated in this study have been found in a variety of other habitats, such as corals (Brochier-Armanet et al., 2008), hot springs (Zhang et al., 2008), estuaries (Santoro et al., 2008), marine sediments (Francis et al., 2005), and soil (Onodera et al., 2010). In a previous 16S rRNA gene based amplicon sequencing study of the same microbial mats (Bolhuis and Stal, 2011) a small number of AOB sequences (less than 0.01% of the total community) related to the Nitrosomonadales was found. In two metagenomic datasets of these microbial mats the AOB genus *Nitrosococcus* and *Nitrosomonas* were found at ~0.5 and 0.2% of the total identified genus, respectively (unpublished). In none of these studies AOA sequences were found. However, due to the different nature of the experiments in the studies cited above and in the present study (DNA hybridization vs. PCR bases gene amplification vs. whole genome sequencing) a comparison between these studies is difficult. The high diversity of ammonia oxidizers that we observed can be explained by the large variety of potential microhabitats in the microbial mats that are generated by the daily fluctuations of geochemical parameters and by the activity of the mat microbes.

The richness and diversity estimators (Shannon-Weaver) indicated that Station 1 harbored a more diverse ammonia-oxidizing community than the other two stations. Cluster analysis showed that the bacterial and archaeal *amoA* composition both grouped Station 2 and Station 3 apart from Station 1 (Figure 2). MRPP analysis of the ammonia oxidizer community (AOB and AOA) confirmed that Station 2 was more similar to Station 3 than either of these stations to Station 1. This agrees with what was found for the community of denitrifiers (*nirS* and *nirK*) in these mats (Fan et al., 2015) and for the community of nitrogen fixers (*nifH*) (Severin et al., 2012). This suggests that the community composition of the different functional groups of microorganisms involved in the nitrogen cycle is determined by the same physical and geochemical factors that prevail in the three types of microbial mats. Previous studies of the eukaryal, bacterial and archaeal diversity (based on ribosomal RNA genes) of these mats revealed that community composition and microbial diversity were intrinsic of the mat type (Bolhuis and Stal, 2011; Bolhuis et al., 2013). Here we show that this also applies for a functional gene for ammonia oxidation.

The richness and diversity of bacterial *amoA* showed more seasonal changes than those of the archaeal *amoA*, with the exception of Station 2. The fluctuation of richness and diversity for archaeal *amoA* at Station 2 may be due to the tidal inundation by seawater. We expected that the richness and diversity might be higher in winter because other studies showed that AOA were more abundant in winter in the North Sea (Wuchter et al., 2006). However, our results showed that this scenario did not occur in the coastal microbial mats. Cluster analysis showed that the AOB composition at Station 2 was more affected by seasonal changes. The possible explanation is that the mat is disappearing at Station 2 during the year, while the other mats are perennial.

AOB *amoA* copy numbers were similar to those reported from estuaries (Caffrey et al., 2007; Santoro et al., 2008), salt marshes (Dollhopf et al., 2005) and coastal aquifer sediments (Mosier and Francis, 2008), whereas AOA *amoA* copy numbers in our study were comparable with those in the cold seep surface sediment (10^2^–10^4^ copies g^−1^ sediment) (Dang et al., 2010) but were two to three orders of magnitude lower than reported for other estuarine and coastal sediments (10^4^–10^7^ copies g^−1^ sediment) (Mosier and Francis, 2008; Santoro et al., 2008). The *amoA* copy number of neither AOB nor AOA correlated with the potential rate of nitrification. This has also been found in other studies (Caffrey et al., 2007; Santoro et al., 2010; Wankel et al., 2011). For instance, Caffrey et al. (2007) did not find a correlation between the number of AOA in the sediment and the potential nitrification rate at four out of six sites in an estuary. Likewise, Santoro et al. (2010) did not find a correlation of either AOB or AOA *amoA* abundance with nitrification rate in the central California Current. The lack of a correlation may have various reasons. Firstly, it is possible that different types of ammonia-oxidizers have different potential rates of ammonia oxidation per cell (Santoro et al., 2010). It is not precisely known whether certain phylotypes, defined by their 16S rRNA gene sequence, share the same physiological characteristics (Prosser and Nicol, 2012). Secondly, it is possible that only part of the nitrifying populations was active and responsible for the nitrification in microbial mats. The lack of positive correlation between AOB *amoA* gene abundance and its transcripts in this study supports the explanation that only part of the nitrifying community was active. Moreover, there is evidence that AOB and AOA are capable of mixotrophic (Qin et al., 2014) or heterotrophic growth (Jia and Conrad, 2009), thus the presence of AOB or AOA does not necessary mean that they oxidize ammonia. In addition, the function of archaeal ammonia monooxygenase is not clear (Prosser and Nicol, 2012). Therefore, AOB or AOA abundance and diversity should not be considered as a proxy for nitrification. Ammonia oxidizers that belong to the Gammaproteobacteria might also contribute to nitrification (Lam et al., 2007). However, the GeoChip showed a negligible signal for nitrifiers of the Gammaproteobacteria when compared to β-AOB and AOA. Therefore, it was concluded that it is unlikely that Gammaproteobacteria play a role of importance in ammonia oxidation in the studied microbial mats.

Some studies reported that AOA are more abundant than AOB in marine (Mincer et al., 2007) and terrestrial ecosystems (Leininger et al., 2006; Shen et al., 2008). Meanwhile, mounting evidence from various estuarine and coastal studies suggested that in certain regions AOB *amoA* gene abundance is higher than that of AOA *amoA* (Caffrey et al., 2007; Mosier and Francis, 2008; Santoro et al., 2008; Wankel et al., 2011). We show that in coastal microbial mats AOB were two to four orders of magnitude more abundant than AOA. On the one hand, AOA *amoA* was not expressed at detectable levels in the microbial mats despite the high diversity of the gene. On the other hand, the AOB *amoA* transcripts positively correlated with potential nitrification rates (Pearson, *r* = 0.638, *p* < 0.05, *n* = 13). Moreover, multiple stepwise linear regressions showed that β-AOB *amoA* transcription was the only valid predictor (out of the variables measured in this study) of the rate of potential nitrification (*r*^2^ = 0.516, *p* < 0.05; r for the whole model). AOB *amoA* transcripts variation explained 51% of the rate of potential nitrification. This evidence suggests that AOB are predominantly responsible for nitrification in the microbial mats investigated in this study.

A study on the archaeal diversity in the same mats as in this study revealed that sequence reads assigned to *Thaumarchaeota* were present in low numbers, hence, confirmed the low abundance of this group in the mats (Bolhuis and Stal, 2011). There are several explanations for the minor importance of AOA in nitrification in the mat. Firstly, salinity appears to play a role in the relative distribution of AOA and β-AOB. Mosier and Francis (2008) found that in coastal aquifer sediments with high salinity (22–31 psu) and low (2–15 μM) ammonia concentration AOB were more abundant than AOA but that at low salinity (0.2–9 psu) the latter prevailed. A similar study across a groundwater seawater beach interface also revealed that AOB *amoA* abundance exceeded AOA *amoA* abundance with proximity to the ocean and higher salinity (Santoro et al., 2008). The salinities of the microbial mats in this study were generally polyhaline. Secondly, the pore water of the mat contains relatively high ammonium concentrations. The majority of AOA found in this study belonged to *Nitrosopumilus*. This lineage is represented by *Nitrosopumilus maritimus*, which appears to be adapted to growth at low ammonia concentrations (Martens-Habbena et al., 2009). This may also be the case for AOA in our mats, although some *Nitrososphaera* strains tolerate higher ammonium concentrations (Verhamme et al., 2011). It is possible that AOA are not obligate ammonia-oxidizers and this would explain the positive correlation between abundance of AOA *amoA* gene and organic matter (Mußmann et al., 2011). Alves et al. (2013) showed that AOA belonging to *Nitrososphaera* are functional heterogeneous and that some would not exclusively grow at the expense of ammonia oxidation.

Constrained correspondence analysis showed that the community composition of both AOA and AOB was influenced by the same factors: salinity, temperature and DIN. The lower salinity at Station 1 may explain the high diversity and distinct ammonia oxidizer community compared to the other two stations. Bernhard et al. (2010) observed that the loss of diversity of ammonia-oxidizing bacteria correlated with increasing salinity in the Plum Island Sound estuary. Salinity influenced not only the composition of the ammonia-oxidizing community but also the abundance of ammonia oxidizers. In this study, AOA and β-AOB *amoA* abundance were both significantly and positively correlated with salinity. The literature does not reveal consistent conclusions with respect to the effect of salinity on ammonium oxidizers. In San Francisco Bay, AOA *amoA* abundance was negatively correlated with salinity and β-AOB *amoA* abundance was positively correlated with salinity (Mosier and Francis, 2008). However, Caffrey et al. (2007) reported that AOA *amoA* abundance was positively correlated with salinity, while no correlation was observed between AOB *amoA* abundance and salinity. These discrepancies indicate that the factors that control the ammonia oxidizer community and *amoA* abundance are complex and not well understood. Salinity alone does certainly not explain the observations sufficiently.

Ammonium concentration is a crucial factor that may determine the community composition of ammonia oxidizers because they differ largely in affinity and tolerance toward ammonia (Prosser and Nicol, 2012). For instance, ammonium concentration influenced AOB community composition and AOB abundance more than in the case of AOA. Particularly, the relative contribution of *Nitrosospira* cluster B [is related to *Nitrosospira* cluster 3 as described by Purkhold et al. (2003)] is positively correlated with ammonium concentration (Pearson, *r* = 0.95, *p* = 0.01, *n* = 5). This is in line with the observation that *Nitrosospira* cluster 3 responded best to high ammonia concentration (Verhamme et al., 2011). The two dimensions of CCA explained only part of the total variance of the ammonia oxidizer community. This implied that other factors must be involved. Many physical and geochemical factors have been proposed including oxygen availability, sulfide concentration (Joye and Hollibaugh, 1995), light (Horrigan and Springer, 1990), and trace metal availability (Mosier and Francis, 2008). All those factors are important in microbial mats but were not taken into account in this study.

The seasonal patterns of potential nitrification observed in the three stations were similar. The low potential nitrification rates in July may be due to competition for ammonium between ammonia oxidizers and cyanobacteria that use it as nitrogen source (in Station 2 also diatoms may compete for the ammonia). Cyanobacteria are the main structural component of these coastal microbial mats. The microbial mat reaches maturity in summer and becomes less productive and the standing stock biomass decreases afterwards (Stal et al., 1985). Nitrification is an aerobic process and therefore in summer can only happen in the light when the cyanobacteria evolve oxygen as the result of photosynthesis. However, the cyanobacteria then also fix CO_2_ and assimilate ammonium for growth (Stal, 2003). Hence, the competition pressure in summer may lead to the lower potential nitrification rate compared to other seasons, when the mats are less active and presumably do not become anaerobic. Also in the coastal Arctic Ocean potential nitrification rates were higher in winter than in summer (Christman et al., 2011). These authors hypothesized that the lack of competition for ammonium with phytoplankton and other microorganisms would stimulate nitrification in winter. Also in line with this was the conclusion of Risgaard-Petersen et al. (2004) that benthic algae are superior to AOB when it comes to competition for ammonium. The seasonal pattern of potential nitrification rates may also be attributed to the dynamics of the community composition of ammonia oxidizers. CCA indicated a seasonal trend for both bacterial and archaeal ammonia oxidizers that correlated particularly to the concentrations of ammonium and nitrate/nitrite. We observed signal intensity shifts in the GeoChip for different types of bacterial ammonia oxidizers between July and January. Because different ammonia oxidizers will have different physiological characteristics, the shift in the community composition may eventually result in the seasonality of nitrification.

## Author contributions

HF designed and carried out the experiments, interpreted the data and wrote the manuscript. HB designed the experiments, interpreted the data and wrote the manuscript. LS designed the project, supervised the experiments, wrote the manuscript.

### Conflict of interest statement

The authors declare that the research was conducted in the absence of any commercial or financial relationships that could be construed as a potential conflict of interest.
